# Appearance of a double bubble in achalasia cardia: a case report

**DOI:** 10.1186/1752-1947-2-383

**Published:** 2008-12-13

**Authors:** Shaheen E Lakhan, S Jeevan Kumar, P Ratnakar Kini

**Affiliations:** 1 Global Neuroscience Initiative Foundation, Los Angeles, CA, USA

## Abstract

**Introduction:**

Achalasia cardia is characterized by failure of the lower esophageal sphincter to relax in response to swallowing and by an absence of peristalsis in the esophageal body. Absence of a gastric air bubble is a well known radiological finding. Pneumatic balloon dilatation results in reappearance of the gastric bubble.

**Case presentation:**

We report the case of a 43-year-old Indian man with achalasia cardia whose chest X-ray at the time of presentation showed an air bubble in the gastric region causing a diagnostic quandary. Successful dilatation of the lower esophageal sphincter resulted in the appearance of another air bubble in the gastric region. Proper analysis showed that the first bubble was actually a colonic air bubble of the splenic flexure and the appearance of the second bubble was the anticipated gastric air bubble.

**Conclusion:**

In patients presenting with achalasia cardia, a colonic air bubble may be seen in the gastric region causing diagnostic difficulty. In these patients, a gastric air bubble may appear after pneumatic dilatation. At the end of the procedure, there will be two air bubbles ("double bubble"): a colonic and a gastric air bubble. To our knowledge, this finding has not been reported in the literature thus far.

## Introduction

Achalasia cardia is characterized by failure of the lower esophageal sphincter to relax in response to swallowing and by an absence of peristalsis in the esophageal body. Absence of a gastric air bubble is a well known radiological finding in patients with achalasia cardia.

## Case presentation

**Figure 1:**
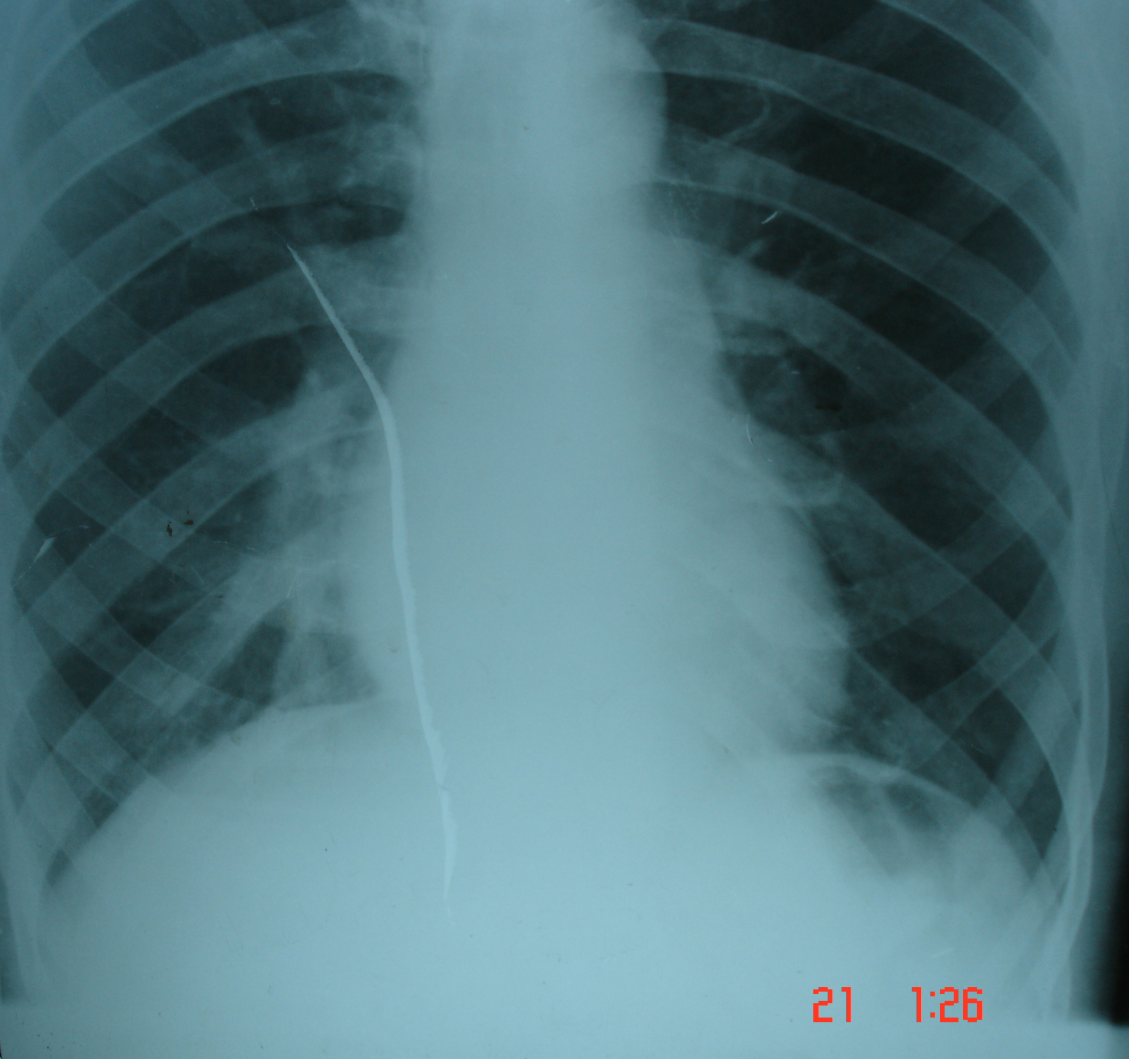
Chest X-ray showing air bubble below the left hemi-diaphragm in the gastric region.

**Figure 2:**
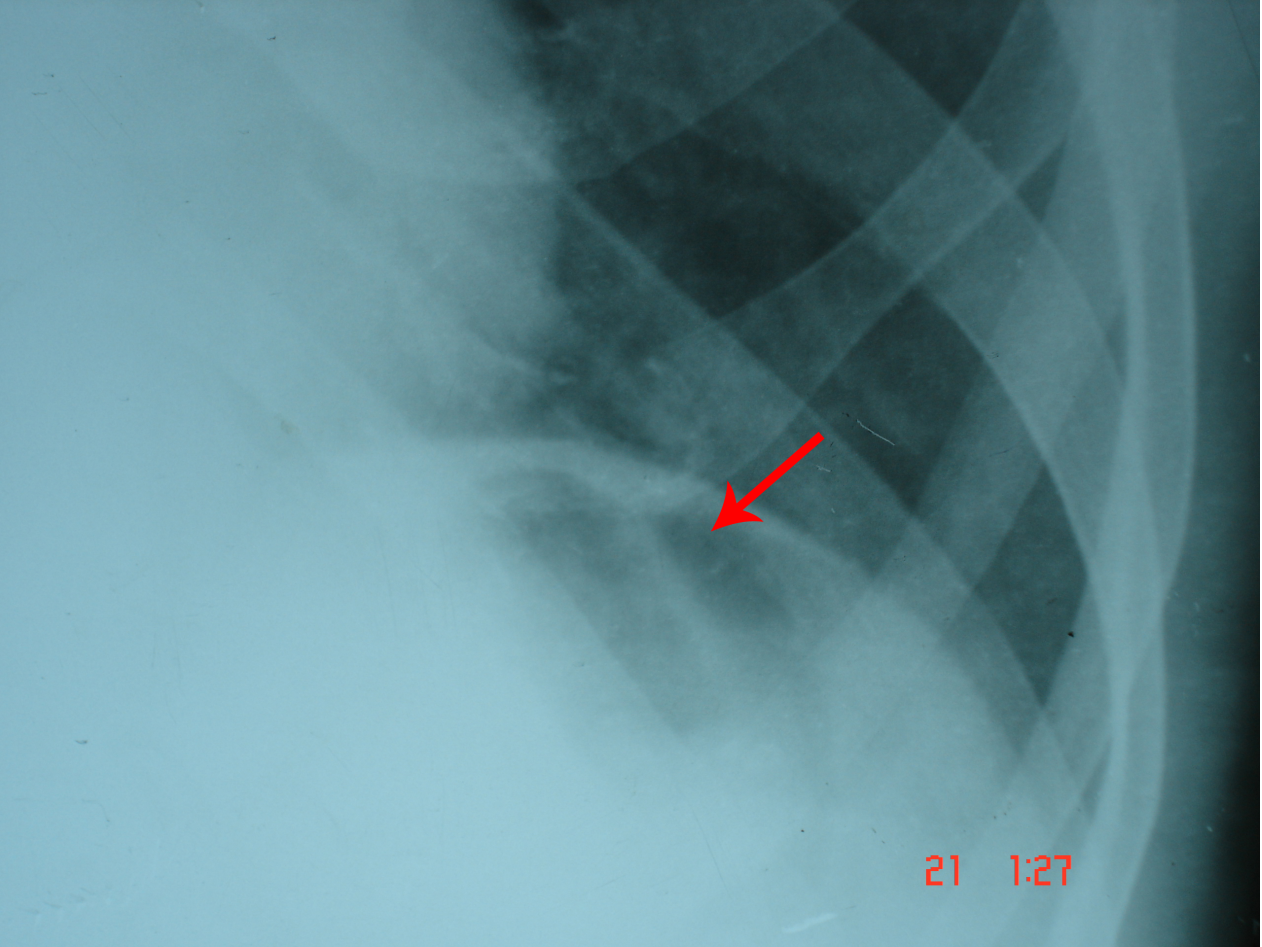
Chest X-ray; red arrow shows the air bubble below the left hemi-diaphragm before pneumatic balloon dilatation. This is an atypical finding.

A 43-year-old Indian man was referred to our gastroenterology department with complaints of dysphagia for both solids and liquids. The symptom was non-progressive. He also had recurrent vomiting and he regurgitated undigested food. He had considerable weight loss. The physical examination was unremarkable. Ultrasound scan of the abdomen showed normal findings. Upper gastrointestinal endoscopy showed a dilated esophageal body and no peristalsis was seen. The lower esophageal sphincter was tightly closed. With gentle pressure, the endoscopist was able to negotiate the endoscope into the stomach. Retroflexion of the endoscope revealed no mass lesion in the esophagogastric junction or in the cardia. All of these features pointed towards the diagnosis of primary achalasia cardia. Chest X-ray of the patient showed an air bubble below the left hemi-diaphragm in the gastric region which is not expected in a case of achalasia cardia (Figures 1 and 2). Barium swallow in this patient showed dilated esophagus, "bird beak" appearance of the distal esophagus and an esophageal air fluid level.

The presence of an air bubble below the left hemi-diaphragm in the gastric region as seen in the chest X-ray posed a diagnostic challenge. But since the clinical history, examination, upper gastrointestinal endoscopy and barium swallow X-ray were suggestive of achalasia cardia, a final diagnosis of achalasia cardia was made and pneumatic balloon dilatation of the lower esophageal sphincter was planned.

Under sedation, pneumatic balloon dilatation of the lower esophageal dilatation was carried out. The procedure was performed under endoscopic vision. The balloon was placed across the lower esophageal sphincter and inflated. The balloon was kept in the inflated position for 2 minutes. With the help of the retroflexed endoscope, active bleeding was seen across the chest junction which indicated a successful dilatation of the lower esophageal sphincter. There were no procedure-related complications.

**Figure 3:**
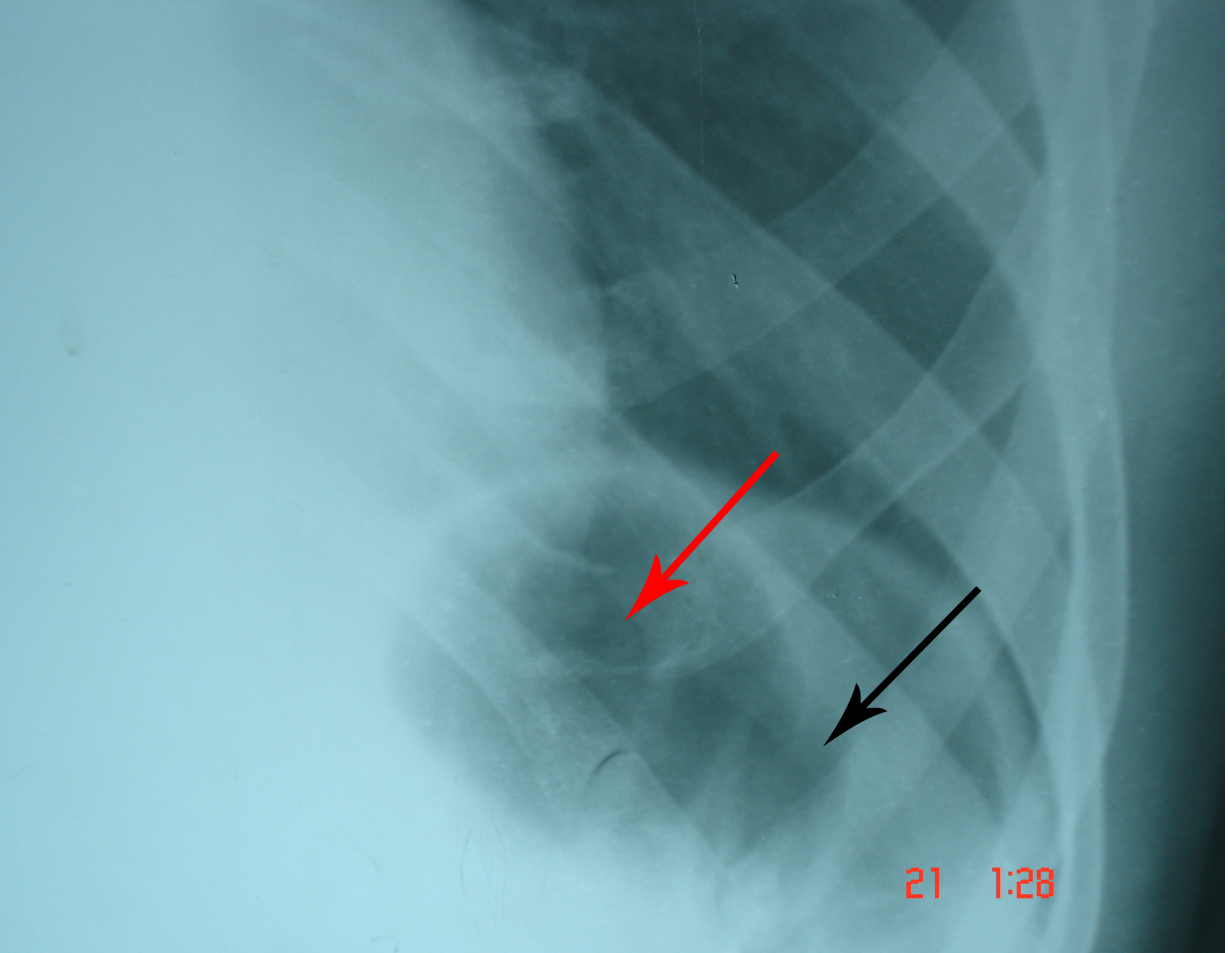
Chest X-ray showing the double bubble after successful dilatation of the lower esophageal sphincter. Red arrows indicate the presence of air in the colon. Note the haustrations. Black arrow indicates the presence of air in the stomach which appeared after dilatation.

A chest X-ray after the procedure showed two air bubbles under the left hemi-diaphragm in the gastric region. A second air bubble had appeared adjacent to the previous one which was present before dilatation. Since a successful dilatation of the lower esophageal sphincter results in the appearance of the gastric bubble, the second air bubble that appeared was the gastric air bubble. Careful examination of the first air bubble present before the procedure, showed haustral markings. This confirmed that the air bubble was a colonic air bubble. The "double bubble" is thus a colonic air bubble and a gastric air bubble (Figure 3).

The colonic air bubble was seen before the procedure mimicking the gastric air bubble which caused diagnostic confusion. After successful pneumatic dilatation, the gastric air bubble appeared below the left hemi-diaphragm, which is an anticipated event. This appearance of a double bubble in a case of achalasia cardia not only causes diagnostic problems, but is also very unusual and has not been reported in the literature before.

## Discussion

Achalasia is a primary esophageal motility disorder involving the body of the esophagus and lower esophageal sphincter affecting equally both genders and all ages [1]. Although endoscopy is considered to have a poor sensitivity and specificity in the diagnosis of achalasia, it has an important role in ruling out secondary causes of achalasia (i.e. pseudoachalasia). A chest X-ray can give important information. It may show the absence of a gastric air bubble. Barium swallow will show dilated esophagus, "bird beak" appearance of the distal esophagus and an esophageal air fluid level. In up to 20% of achalasia patients, however, these classic X-ray findings are not present. Manometry is the gold standard for diagnosing achalasia cardia [1]. In patients with typical radiographic findings of achalasia, the barium study can be used to guide treatment without a need for manometry. If radiographic findings are equivocal, however, manometry may be required for a more certain diagnosis [2]. But manometry is not available in all medical centers. In centers where manometry is not available, clinical history, endoscopy, chest X-ray and barium swallow are all taken together to diagnose achalasia cardia. With respect to treatment, Heller's myotomy and pneumatic balloon dilatations of the lower esophageal sphincter are considered definitive treatments for achalasia [3].

Since achalasia cardia is associated with failure of the lower esophageal sphincter to relax, not enough air passes across into the stomach. This is manifested as an absent gastric bubble in the abdominal X-rays. Though this is not a sensitive method, absence of a gastric air bubble in the chest X-ray is one of the significant findings for diagnosing achalasia [4]. After successful dilatation of the lower esophageal sphincter, the gastric air bubble reappears in the chest X-ray.

Sometimes achalasia presents with atypical presentations and atypical findings. There are case reports of achalasia presenting as acute airway obstruction and recurrent pneumonitis [5,6]. In patients with atypical presentation and findings, the diagnosis is often delayed. In our patient, there was an atypical finding in the form of the presence of an air bubble below the left hemi-diaphragm in the gastric region in the chest X-ray film.

Based on the clinical history, examination, upper gastrointestinal endoscopy and barium swallow X-ray findings, a provisional diagnosis of achalasia cardia was made. Pneumatic balloon dilatation was done to relieve the symptoms. The chest X-ray taken after the successful procedure showed the appearance a second air bubble in the gastric region adjacent to the previous one. This phenomenon is an anticipated one. Careful examination of the first air bubble, which was seen even before the dilatation was done, showed haustral markings. Haustral markings are seen in the colon. This led us to the conclusion that the air bubble which was present before dilatation was indeed a colonic air bubble in the splenic flexure. Therefore, the second air bubble, which appeared after successful dilatation of the lower esophageal sphincter, was the gastric air bubble.

So in our patient, at the end of the dilatation, there were two air bubbles – a double bubble. A thorough Medline search was performed. To our knowledge, this finding has not been reported in the literature thus far. The appearance of a double bubble in patients with achalasia cardia is an interesting finding following a successful dilatation of the lower esophageal sphincter. This double bubble sign may pose a diagnostic challenge in the patients in whom it is present. Knowledge of this unusual sign may be helpful in these circumstances.

## Conclusion

A colonic air bubble in the splenic flexure may mimic a gastric air bubble in chest X-ray films. This may cause confusion in the diagnosis of achalasia cardia where the gastric bubble is generally absent. Successful dilatation of the esophageal sphincter in patients with achalasia cardia results in reappearance of the gastric air bubble. In patients whose chest X-ray shows a colonic air bubble in the gastric region at the time of presentation, the chest X-ray will show a double bubble after successful dilatation of the lower esophageal sphincter. The double bubble represents the colonic air bubble and the gastric air bubble.

## Consent

Written informed consent was obtained from the patient for publication of this case report and any accompanying images. A copy of the written consent is available for review by the Editor-in-Chief of this journal.

## Competing interests

The authors declare that they have no competing interests.

## Authors' contributions

SL, SJK, and PRK secured the case, conducted the literature review, and participated in the preparation of the manuscript. All authors read and approved the final manuscript.

## References

[B1] Vaezi MF Richter JE Diagnosis and management of achalasia. American College of Gastroenterology Practice Parameter Committee Am J Gastroenterol 1999 94 12 3406 3412 10.1111/j.1572-0241.1999.01639.x 10606295

[B2] Amaravadi R Levine MS Rubesin SE Laufer I Redfern RO Katzka DA Achalasia with complete relaxation of lower esophageal sphincter: radiographic-manometric correlation Radiology 2005 235 3 886 891 10.1148/radiol.2353040732 15833990

[B3] Pohl D Tutuian R Achalasia: an overview of diagnosis and treatment J Gastrointestin Liver Dis 2007 16 3 297 303 17925926

[B4] Orlando RC Call DL Bream CA Achalasia and absent gastric air bubble Ann Intern Med 1978 88 1 60 61 619760 10.7326/0003-4819-88-1-60

[B5] Khan AA Shah SW Alam A Butt AK Shafqat E Malik K Amin J Achalasia esophagus; presenting as acute air way obstruction J Pak Med Assoc 2007 57 8 423 425 17902530

[B6] Sharma GL Kumar A Mukund A Kedia A Atypical presentation of achalasia cardia: a case report Indian J Radiol Imaging 2005 15 175 176

